# Medullary Thyroid Cancer: Updates and Challenges

**DOI:** 10.1210/endrev/bnad013

**Published:** 2023-05-19

**Authors:** Matti L Gild, Roderick J Clifton-Bligh, Lori J Wirth, Bruce G Robinson

**Affiliations:** Faculty of Health and Medicine, University of Sydney, Sydney 2006, Australia; Department of Diabetes and Endocrinology, Royal North Shore Hospital, Sydney 2065, Australia; Cancer Genetics, Kolling Institute of Medical Research, Sydney 2065, Australia; Faculty of Health and Medicine, University of Sydney, Sydney 2006, Australia; Department of Diabetes and Endocrinology, Royal North Shore Hospital, Sydney 2065, Australia; Cancer Genetics, Kolling Institute of Medical Research, Sydney 2065, Australia; Department of Medicine, Massachusetts General Hospital, & Harvard Medical School, Boston 02114, USA; Faculty of Health and Medicine, University of Sydney, Sydney 2006, Australia; Department of Diabetes and Endocrinology, Royal North Shore Hospital, Sydney 2065, Australia; Cancer Genetics, Kolling Institute of Medical Research, Sydney 2065, Australia

**Keywords:** tyrosine kinase inhibitor, multiple endocrine neoplasia, calcitonin‌

## Abstract

A personalized approach to the management of medullary thyroid cancer (MTC) presents several challenges; however, in the past decade significant progress has been made in both diagnostic and treatment modalities. Germline rearranged in transfection (RET) testing in multiple endocrine neoplasia 2 and 3, and somatic RET testing in sporadic MTC have revolutionized the treatment options available to patients. Positron emission tomography imaging with novel radioligands has improved characterization of disease and a new international grading system can predict prognosis. Systemic therapy for persistent and metastatic disease has evolved significantly with targeted kinase therapy especially for those harboring germline or somatic *RET* variants. Selpercatinib and pralsetinib are highly selective RET kinase inhibitors that have shown improved progression-free survival with better tolerability than outcomes seen in earlier multikinase inhibitor studies. Here we discuss changes in paradigms for MTC patients: from determining *RET* alteration status upfront to novel techniques for the evaluation of this heterogenous disease. Successes and challenges with kinase inhibitor use will illustrate how managing this rare malignancy continues to evolve.

## Graphical Abstract

**Graphical Abstract:**
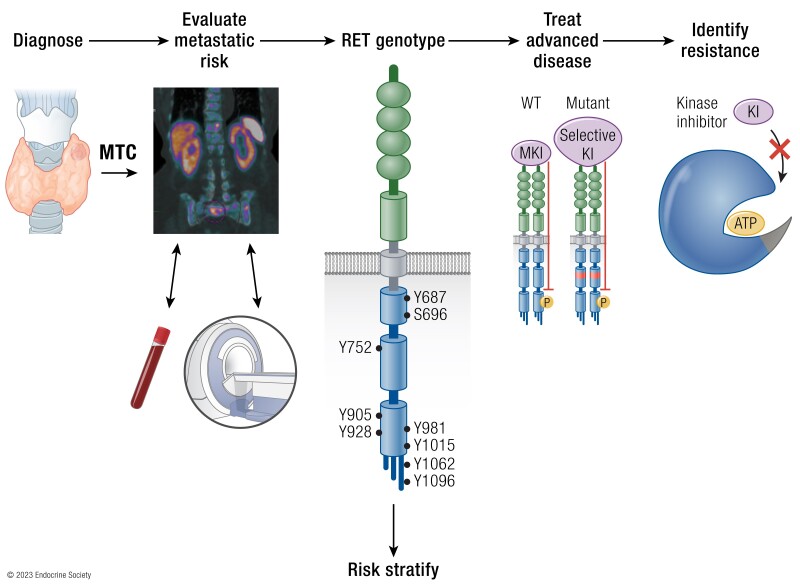

**Essential Points**

- Up to 25% of medullary thyroid cancer (MTCs) occur in the context of well-defined familial endocrine syndromes with autosomal dominant inheritance, as such all patients with MTC require germline *RET* mutation analysis
- Positron emission tomography imaging with novel radioligands (^18^F-DOPA and ^68^Ga-DOTATATE) can be used for enhanced detection of both recurrent and metastatic MTC. ^18^F-DOPA has a higher sensitivity of lesion detection
- RET-specific kinase inhibitors (selpercatinib and pralsetinib) have the benefit of potent inhibition of the oncogenic driver with less off-target kinase inhibition and better patient tolerability and have shown success in clinical trials
- Acquired resistance to multikinase inhibitors yielding clinical progression is a new challenge in MTC therapeutics. Resistance mechanisms are usually either on target; within the kinase domain itself, or off-target or bypass alterations

A personalized approach to the management of medullary thyroid cancer (MTC) presents several challenges. While MTC only accounts for <5% of thyroid cancers (possibly even less with the incidental incidence of differentiated thyroid cancer on the rise), the value of precise diagnosis and treatment in advanced disease is disproportionate to the prevalence of the disease. This is reflected in its accounting for up to 13% of thyroid cancer deaths (1).

MTC arises from parafollicular C cells within the thyroid, which are of neural crest origin, and hence MTC is considered a neuroendocrine tumor. MTCs are either sporadic or hereditary. They may present in 1 of 5 different ways: most commonly as a thyroid lump (74% of sporadic presentations) (1); as a mass from metastatic disease (cervical lymph nodes or distant metastases); from symptoms secondary to elevated calcitonin (diarrhea, flushing) (10% of sporadic cases) (1); as ectopic Cushing syndrome (0.7%) (2); or detected after familial screening. MTC disproportionately accounts for 10.5% of all thyroid cancers with distant metastases at first presentation, mostly to liver and bone (3). Cytological diagnosis of MTC on fine-needle biopsy presents some challenges for the pathologist: only 56% of MTCs are accurately diagnosed before surgery (4). The addition of preoperative measurement of serum calcitonin improves this diagnostic accuracy, which then ensures appropriate surgical management.

Part of the reason for poor outcomes from MTC is late diagnosis relative to metastatic potential; 70% of patients with MTC who present with a palpable thyroid nodule have cervical metastases and 10% have distant metastases (1). Fortunately, early diagnosis is made possible for familial MTC syndromes. Up to 25% of MTCs occur in the context of well-defined familial endocrine syndromes with autosomal dominant inheritance. Since family history of MTC may be absent in as many as 50% multiple endocrine neoplasia (MEN) type 2 cases (1), all patients with MTC require germline rearranged in transfection (*RET*) mutation analysis. Genotype–phenotype correlations have allowed familial cases with specific *RET* variants to be stratified according to the level of risk. American Thyroid Association (ATA) clinical guidelines can help determine the age at which prophylactic thyroidectomy may be appropriate (Fig. 1) (5).

**Figure 1.**
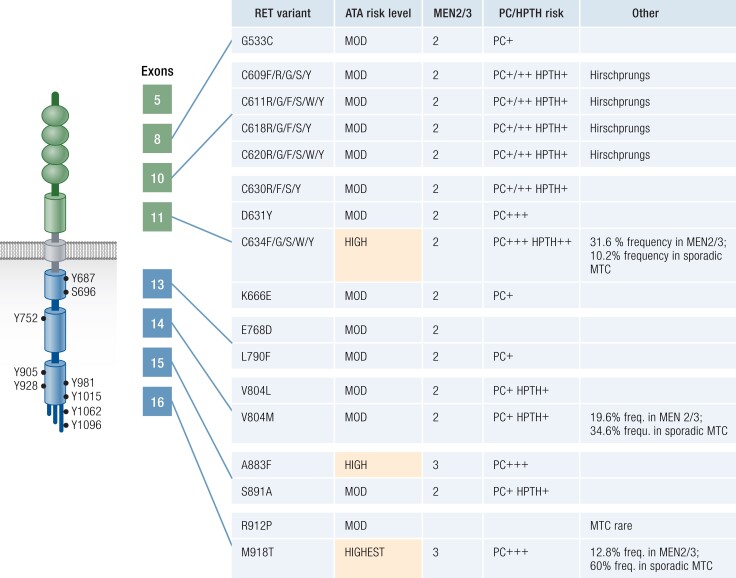
Genotype–phenotype correlations, allelic frequency and risk levels for medullary thyroid carcinoma behavior∗. Adapted from Wells et al, 2015 Revised American Thyroid Association guidelines for the management of medullary thyroid carcinoma (5). Rare mutations based on very few families with genetic variants of unknown significance are not included. These variants by amino acid change are displayed according to abbreviation. Please note HGVS nomenclature now suggests variants be described as in text (ie, M918T as p.MET918Thr). Frequency of variants in MEN2/3 and sporadic MTC sourced (6, 7).

In the past decade significant progress has been made in the diagnostic and treatment approaches to MTC. Positron emission tomography (PET) imaging with novel radioligands has improved characterization of disease. A new international grading system has been proposed (8). Lastly, systemic therapy for persistent and metastatic disease has evolved significantly over the last decade with targeted kinase therapy especially for those harboring germline or somatic *RET* variants. Evidence from phase II and III trials showed improved progression-free survival (PFS) with multikinase inhibitors (MKIs) such as vandetinib, cabozantinib, and lenvatinib, but often with dose-limiting toxicities (9-11). Recently, highly selective RET kinase inhibitors (selpercatinib and pralsetinib) have shown improved PFS with better tolerability than outcomes seen in earlier MKI studies (12, 13).

In this review we discuss changes in paradigms for MTC patients, from determining *RET* alteration status upfront to novel techniques for the evaluation of this heterogenous disease. We also highlight the successes and challenges with kinase inhibitor use to illustrate how managing this rare malignancy continues to evolve.

## Prevalence and Epidemiology

The incidence of MTC varies across the world. In China the incidence has decreased in the 21st century possibly due to a combination of recording errors from an absence of MTC population databases and less genetic screening programs (14). Approximately 1200 new MTC patients are diagnosed each year in the United States (15). The age-adjusted prevalence of MTC has increased with an average annual percent change of 1.87% between 1993 and 2012 (16). The cause of this reported increased incidence is not well understood. Obesity has been identified as a contributing factor to an overall increase in thyroid cancer prevalence of all subtypes except medullary (16). MTC is usually reported as <5% of thyroid cancers but, the disproportionate increase in incidence of papillary thyroid cancer has resulted in the SEER database reporting it as only 1% and 2% of thyroid cancer (5). The 10-year survival rates for distant metastatic MTCs are 21% (5).

## Pathogenesis

### C Cell Hyperplasia

MTC is derived from the parafollicular “C” cells in the thyroid gland, which originate from the neural crest. C cell hyperplasia may sometimes be physiologic, but when seen in the setting of familial disorders, it is considered premalignant, and sometimes referred to as “in situ MTC” (17). When developing secondary to autosomal dominant familial pathway alterations, it is usually located laterally in the upper two-thirds of the thyroid with histological atypia (18). C cells produce calcitonin which is a reasonably sensitive marker of MTC, but its specificity is limited since it can be elevated in other malignant (carcinoid and small cell lung cancers) and nonmalignant conditions (hypercalcemia, pregnancy, renal failure, sepsis, pheochromocytoma, autoimmune thyroiditis) (19). After thyroidectomy, specificity is improved and calcitonin becomes central to MTC follow up management (discussed below).

### Rearranged in Transfection

The RET proto-oncogene (summarized in (20)) encodes for a receptor tyrosine kinase upstream of several established pathways including include phospholipase Cγ/protein kinase C, c-Jun N-terminal kinases, and the established oncogenic pathways including products of the proto-oncogene Src-related kinases phosphatidyl-inositol-3-kinase and mitogen-associated protein kinase. Pathogenic *RET* variants in MTC are usually point mutations which induce constitutive activation of RET through phosphorylation (20). Mutations in the cysteine rich domain cause a breakdown of intrachain disulfide bonds, which then reconstruct disulfide bonds resulting in RET homodimerization. This leads to uncontrolled activation of the abovementioned downstream pathways causing amplification of cellular proliferation, and changes in migration and differentiation.

### Sporadic vs Hereditary MTC

Up to 25% of MTCs occur as part of a hereditary syndrome and 75% are sporadic. Hereditary MTC occurs in MEN syndromes type 2 and 3 (formerly 2B), and familial MTC, which is now considered a subset of MEN2. These are all autosomal dominant syndromes caused by pathogenic germline variants in the *RET* proto-oncogene. Similar somatic *RET* alterations are also found in around 60% of sporadic MTCs (21); many of the remainder of sporadic MTC are driven by somatic mutations in *RAS*, of which *HRAS* p.Gln61Arg is the most frequent (21). It is generally accepted that *RAS*-mutated MTCs behave less aggressively (22) than those harboring somatic *RET* p.Met918Thr alterations (23). The *RET* p.Met918Thr alteration is the most common alteration found in sporadic MTC, but mutations in multiple other codons (883, 634) and deletions have also been described (20). In 1 meta-analysis, the presence of a *RET* alteration in sporadic MTC was associated with an elevated risk for lymph node metastasis, advanced tumor stage and tumor recurrence (24). In contrast, a more recent meta-analysis showed no association with recurrence risk (25).

### RET Variants

*RET* is well characterized and pathogenic *RET* variants are tightly clustered; hence, genetic analysis can be confined to specific exons. Numerous variants have been identified in multiple exons (10, 11, 13, 14, 15, 16, and rarely 1, 5, 8) (20, 26, 27). Cysteine-encoding codons 609, 611, 618, 620, or 634 are commonly altered in MEN2, with arginine substitution at codon 634 the most common (Fig. 1). The loss of these extracellular cysteine residues leads to ligand-independent dimerization and constitutive activity (28). In contrast, the most common alteration in MEN3, p.Met918Thr, lies within the intracellular tyrosine kinase domain. This alteration leads to constitutive kinase activation and oncogenic pathway signaling (29).

The prevalence of *RET* indels is variable but may be up to 17% in a large series of patients with RET-mutated sporadic MTC (30). These are usually located within exon 11 or 15 and have been demonstrated in silico to be “disease causing.” Patients harboring more than 1 *RET* mutant together with indels or with indels alone present with more aggressive disease (30).

### Multiple Endocrine Neoplasia Type 2

MEN is defined by the occurrence of tumors involving 2 or more endocrine glands within a single patient. MEN2 accounts for the majority (95%) of the hereditary cancer syndromes associated with MTC. In MEN2, although MTC is usually the first tumor to develop, 50% of patients will also develop pheochromocytomas and 20% and 30% primary hyperparathyroidism (31). Penetrance for MTC in MEN2 has been reported at 70% at 70 years of age, and 90% of MEN2 carriers will eventually develop MTC irrespective of variant (31, 32). Crucially, there is a strong genotype–phenotype relationship, particularly for age of onset and overall penetrance, and these data help determine timing of risk-reducing thyroidectomy as per ATA guidelines (Fig. 1) (5).

### Multiple Endocrine Neoplasia Type 3

MEN3 (formerly MEN2B) is a more aggressive phenotype with a higher penetrance and very early–onset MTC (>90%) (33). The phenotype includes MTC and pheochromocytoma (but not hyperparathyroidism) together with Marfanoid habitus, mucosal neuromas, medullated corneal fibers, and intestinal autonomic ganglion dysfunction, often leading to multiple diverticulae and megacolon (34, 35). In MEN3, MTC often presents in infancy and can metastasize early. Nearly all MEN3 occurs de novo, but for familial cases diagnosed with germline *RET* p.Met918Thr, risk-reducing thyroidectomy is recommended in the first year of life (5). While 95% of MEN3 cases are due to germline *RET* p.Met918Thr, 5% are associated with *RET* p.Alal883Phe, and this cohort may fare better clinically with a more indolent course of MTC (36).

### Genetic Counselling and Testing

The genotype–phenotype association in hereditary MTC has been well described from the International RET mutation consortium analysis (37). Stratification of risk is dependent on which *RET* alteration is present, and as such there are clear guidelines for timing risk-reducing thyroidectomy to reduce MTC mortality in asymptomatic *RET* carriers (5). As we become more informed about the prevalence of certain variants in the general population via well-curated databases, there may be some adjustment to managing low penetrant *RET* variants such as p.Val804Met (Fig. 1). For instance, only 51% carriers of *RET* p.Val804Met in a German cohort went on to develop MTC, raising the question whether close clinical surveillance was preferrable to risk-reducing thyroidectomy in this circumstance (38). p.Val804Met is the most common pathogenic RET variant seen in large population databases such as gnomAD (39), and is likely to be sub penetrant, such that this variant is over-represented in MEN2 in patients without a family history, whereas when MEN2 is associated with a clear family history of MTC then p.Cys634Arg is the most common variant detected (7).

All patients with MTC should undergo germline *RET* testing of exons 8, 10, 11, 13-16 to exclude MEN2/3. Even in the absence of a family history, germline testing of apparently sporadic MTC results in a diagnosis of MEN2 in 6% cases (40). First-degree relatives should be offered genetic counselling and testing if a germline pathogenic *RET* variant is identified in a patient with MTC. If germline screening is negative, clinicians can be almost certain that the patient does not harbor a hereditary syndrome as 97% of MEN families have well characterized *RET* alterations.

## Tumor Markers

Calcitonin is a highly sensitive tumor marker used in the diagnosis, assessment and follow-up of MTC. C cells appear not to lose their secretory capacity when becoming neoplastic, rendering it an excellent tumor marker except in the very rare cases of nonsecretory MTC (0.83% in 1 MTC cohort). Carcinoembryonic antigen (CEA) is another C cell marker used often in addition to calcitonin. Calcitonin levels may assist with timing of risk-reducing surgery for RET kindreds. In addition, measurement of calcitonin as part of thyroid nodule assessment may be helpful for indeterminate (Bethesda III/IV) nodules but this is not currently recommended in the ATA guidelines. One meta-analysis showed a sensitivity of 100% (83-100%), specificity of 97.2% (94-100%) with a threshold of 10 pg/mL for MTC diagnosis with the caveat of a lack of long-term follow-up data (41). Diagnostic molecular testing for indeterminate nodules have yielded accurate detection of MTC in ThyroSeq v3 and Afirma XA, but cost and availability can limit this resource (42, 43). Preoperative calcitonin levels correlate with the degree of metastatic disease. Levels of <53 pg/mL reflect a low likelihood of lymph node metastases and if over 500 pg/mL, conversely, a high likelihood. At levels of >1000 pg/mL, distant metastatic disease is highly suggestive and preoperative staging should include extensive structural imaging (see below) (19, 44). Surgical management may also be guided by preoperative calcitonin level. When preoperative levels are >200 pg/mL, contralateral neck dissection should be considered in addition to total thyroidectomy, bilateral, and ipsilateral neck dissection, but this is controversial (see “Surgical Management”) (5).

Doubling times (DTs) of calcitonin and CEA are recognized as reliable indicators of disease progression (45). The natural history of MTC varies considerably between patients and can range from indolent to aggressive, hence individual assessment of calcitonin DT is helpful in determining the nature of each MTC. Barbet et al described the prognostic value of calcitonin DT superior to clinical staging, and found all patients with DT >2 years alive at study follow-up (mean 10.5 years) (46). In a small cohort, a short calcitonin DT has been suggested as a trigger to initiate kinase inhibitors in metastatic MTC, but this requires further study (47). The long half-life of calcitonin complicates interpretation of an immediate postoperative value with t_½_ reported around 30 hours (48). As such, calcitonin levels should ideally be reassessed at least 1 month after thyroidectomy (48). Low/normal calcitonin 3 months postoperatively is highly suggestive of complete surgical response (19) and as such intervals for assessment can then be extended.

## Disease Assessment

**Figure 2.**
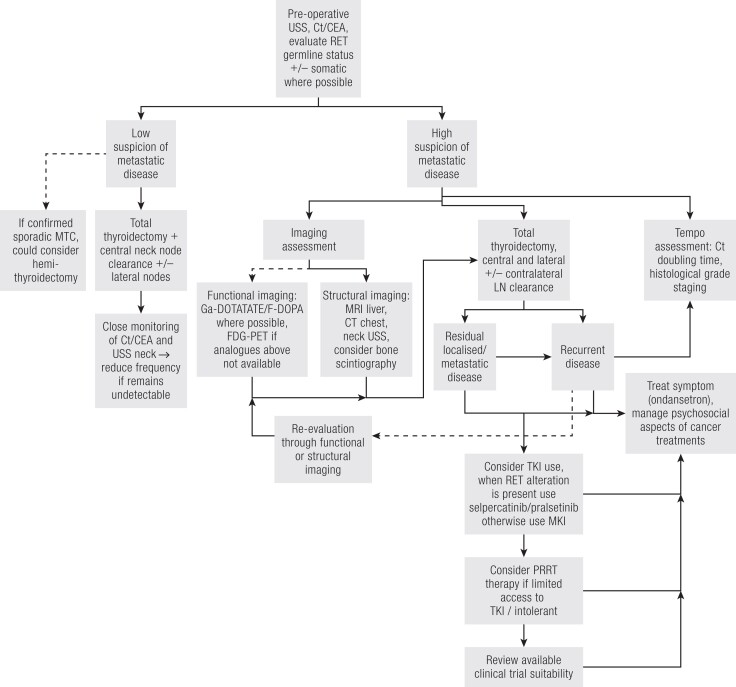
Management algorithm for MTC. Dashed line, alternative option; solid line, recommended; USS, ultrasound; Ct, calcitonin; CEA, carcinoembryonic antigen; MKI, multikinase inhibitor; TKI, tyrosine kinase inhibitor; PRRT, peptide receptor radionuclide therapy; CT, computed tomography; MRI, magnetic resonance imaging.

Initial treatment of MTC is primarily surgical. Undetectable calcitonin implies complete surgical response and the risk of recurrence is low. Clinical review with ultrasound evaluation together with biochemical assessment of calcitonin and CEA yearly is currently recommended (Fig. 2). Persistent elevation of serum calcitonin and CEA are useful markers for the presence of residual or recurrent MTC. Patients may have already presented with distant metastases. The most common sites of distant metastases from MTC are liver (most common), lung, bone, and brain. Regional nodal spread in the neck and chest is very common and can occur early. During follow-up, localization of recurrence may be difficult, particularly when calcitonin or CEA levels are only modestly elevated. A combination of structural and functional imaging is recommended both in primary staging when there is a high risk of metastatic disease and then to evaluate recurrent disease effectively.

## Structural Imaging

Magnetic resonance imaging (MRI) is often employed in structural evaluation for recurrent or metastatic MTC for both staging and then for evaluation of treatment response. Liver metastasis is best assessed with dedicated MRI (49) but clinicians should be aware of potential false positive findings (50). Computed tomography (CT) is preferred for imaging lymph nodes and lung metastases (5). Prior to the widespread use of functional imaging, recommended work-up for metastatic MTC included neck ultrasound, chest CT, liver MRI, bone scan and axial skeleton MRI (51). In recurrent MTC, a rising calcitonin should prompt extensive structural imaging. However, depending on availability of PET imaging and clinician judgement a combination of both modalities may be appropriate.

## Functional Imaging

PET-CT using somatostatin analogs have enhanced detection of both recurrent and metastatic MTC. ^68^Ga-DOTATATE, ^68^Ga-DOTATOC, and ^68^Ga-DOTANOC are radiotracers that have a high affinity for the type 2 somatostatin receptor, expressed in many but not all MTCs. In 1 study of residual and metastatic MTC, ^68^Ga-DOTATATE had a higher lesion detection rate (68.2% vs 44.4%) than ^18^FDG-PET (52). In another analysis of recurrent and metastatic disease, ^68^Ga-DOTATATE was again superior than ^18^FDG-PET (88.1% vs 72.4%) (53). However, ^68^Ga-DOTATATE avidity is often low in metastatic lesions (54). A combination of PET imaging may be complimentary, in that ^68^Ga-DOTATATE can show substantial lesions, whereas ^18^FDG-PET confers better anatomical localization and spatial resolution (Table 1) (55).

**Table 1.** Summary of suitability for radiotracers for medullary thyroid cancer (MTC)

Analog	Positives
**^18^F-DOPA-PET**	Can assist with identification of occult metastatic MTC
**^18^FDG-PET**	Anatomical localization and spatial resolution Positivity associated with reduced survival
**^68^GA-DOTATATE-PET**	Higher sensitivity than ^18^FDG-PET Low prevalence of high tumor avidity in metastatic MTC (50) Better for bone and lymph node assessment compared to ^18^FDG-PET (48)

Differentiating between different PET radiopharmaceuticals for identifying recurrent MTC was examined in a meta-analysis that reviewed 14 trials including 306 patients using ^18^F-FDG, ^18^F-DOPA, ^68^Ga-somatostatin analogs, and C-methionine (56). ^18^F-DOPA had a higher lesions detection rate than ^18^FDG-PET irrespective of calcitonin or CEA DT. In a retrospective comparison with ^68^Ga-DOTANOC, ^68^Ga-DOTATOC, ^18^F-DOPA, and ^18^FDG-PET in searching for residual or recurrent MTC, ^18^F-DOPA showed higher sensitivity (72.2%) than 16.7% for ^18^F-FDG and 33.3% for ^68^Ga-somatostatin analogs (57). ^18^F-DOPA had a particular value for detection of occult metastatic MTC with a low Ki-67 (50) and in fact ^18^FDG-PET together with ^18^F-DOPA is sometimes recommended to identify occult metastases and/or sites of more aggressive disease (CEA DT <24 months) (50). Availability of ^18^F-DOPA is not widespread, particularly in the United States, so in clinical use it is more likely that ^18^FDG-PET may be ordered when required.

Functional imaging has been compared with calcitonin DT and other markers to assess prognosis. Calcitonin and CEA DT (months) did not correlate with the number of lesions detected on ^18^F-DOPA PET/CT (50). Another study showed ^18^FDG-PET positivity was associated with reduced survival but ^18^F-DOPA had a higher sensitivity of lesion detection (58). Of note, when calcitonin DT was >24 months, imaging with either ^68^Ga-DOTANOC, ^68^Ga-DOTATOC, ^18^F-DOPA, or ^18^FDG-PET was negative for all tracers further reflecting the challenge of identifying recurrent indolent disease (57).

Cholecystokinin (CCK) receptor subtype 2 mediates the function of CCK and gastrin. CCKR2 is overexpressed in neuroendocrine tumors and has been detected in MTCs (>90%) (59, 60). Analogs radiolabeled with ^111^In were able to identify occult metastatic MTC lesions but were compromised by nephrotoxicity (60). PET-CT with ^68^Ga-MG48, a gastrin analog, has been compared with ^68^Ga-DOTATATE in lesion assessment (61). Small clinical studies have shown success in theranostics (62), and larger trials are ongoing to evaluate its effectiveness in clinical practice (63).

Selection of the radiopharmaceutical should be based on whether one is aiming to identify residual, recurrent, or metastatic disease as the sensitivities for each modality can vary. There was considerable heterogeneity in many of the comparison studies but taken together there was a trend for a higher sensitivity for ^68^Ga-DOTATATE over ^18^FDG-PET in lesion detection (64). In addition, ^18^F-DOPA PET had the best performance for detection of metastatic MTC on both patient and lesion based analysis in multiple studies. Overwhelmingly though, it is the access to these radiopharmaceuticals that is the limiting factor in deciding which to select.

## Histopathology

Ongoing challenges predicting overall survival in MTC due to its heterogenous nature has been investigated from a histological perspective. Classic cytological MTC criteria on fine-needle aspiration can include a dispersed cell pattern of polygonal or triangular cells, azurophilic cytoplasmic granules, together with eccentrically placed nuclei with coarse granular chromatin and amyloid (5). Controversies regarding the prognostic value of various histological findings including the potentially concerning presence of spindle cell morphology, desmoplasia, mitotic activity, and vascular invasion are widespread in the literature (65). However, a revised histopathological grading providing improved prognostic information was proposed in 2020 (8). Three histological features (Ki-67 proliferative index, mitotic count, and the presence of coagulative necrosis) were rated and then divided into low, intermediate, and high grade which then correlated with prognosis (8). Similarly, this scoring system was applied to microMTC, which showed that it was reproducible (66).

## Surgical Management

The optimal approach to surgery can be nuanced and depend on multiple factors, including comorbidities and extent of disease. Surgical volume is directly correlated with both better short- and long-term outcomes postoperatively. Surgery performed by low/intermediate volume surgeons has been associated with disease recurrence (67). Prior to surgery as part of standard care (if *RET* mutation status is unknown), plasma fractionated metanephrines should be measured to exclude pheochromocytoma. Rarely, MTC is identified in a hemithyroidectomy specimen; completion thyroidectomy is recommended for any patients with germline RET mutations, evidence of residual disease or elevated postoperative calcitonin. Microcarcinomas <1 cm are rarely associated with distant metastases (1.3%), but increasing tumor size is associated with lymph node involvement (5). Overall, 40% to 50% of MTC will present with regional lymph node involvement. For locoregional MTC without distant metastasis, a comprehensive operation including total thyroidectomy and lymph node dissection is usually recommended (5). However, in confirmed sporadic MTC, hemithyroidectomy could be appropriate, yielding no additional recurrence in the preserved lobe in 1 cohort (68) and minimizing operative complications (69). In the absence of cervical nodal disease on ultrasound, and no evidence of distant metastases, ATA guidelines recommend dissection of lateral lymph nodes (levels II-V) to be evaluated based on serum calcitonin levels (Fig. 2) (5). Lateral cervical nodal clearance is recommended when preoperative imaging (ultrasound) of the ipsilateral neck is positive. Contralateral neck dissection remains contentious. ATA guidelines recommend a contralateral lymph node dissection when calcitonin is >200 pg/mL in an effort to achieve cure at initial operation (5); however, the British Thyroid Association guidelines note its impact on survival is less certain (70).

Revision neck surgery may be appropriate for recurrent or metastatic MTC with either curative or palliative intent. Cervical nodal disease and solitary lung metastases are often suitable for excision. If liver metastases are suspected but not visualized on structural or functional imaging, then laparoscopy may be appropriate in select cases (5).

## Multikinase Inhibitors

In the last decade, MKIs have changed the outcomes of patients with advanced MTC resulting in improvement in PFS. Many MKIs, including motesanib, sorafenib, sunitinib, axitinib, imatinib, pazopanib, anlotinib, have been evaluated in MTC (reviewed in (71)) but only vandetanib and cabozantinib have been approved by regulatory authorities for metastatic MTC and, as such, are the MKIs predominantly in clinical use (Table 2) (9, 72).

**Table 2.** Outcomes of kinase inhibitors FDA approved for MTC

Kinase inhibitor	Outcome	% patients requiring dose reduction*^a^*	% patients requiring discontinuation*^a^*
**Vandetinib** p**hase III (**9**)**	Median PFS 30.5 months, HR: 0.46 (0.31-0.69), OS HR: 0.89 (0.48-1.65)	35%	12%
**Cabozantinib** p**hase III (**10**)**	Median PFS 11.2 months, HR: 0.28 (0.19-0.40), median OS 44.3 months, HR: 0.85 (0.64-1.12)	79%	16%
**Selpercatinib** p**hase 1/II (**12**)**	Pretreated patients ORR of 69% (95% CI 55-81%) and 1 year PFS of 82% (95% CI 69-90%) Treatment-naïve patients: ORR 73% (95% CI 62-82%) and 1 year PFS 64% (95% CI 37-92%).	30%	2%
**Praseltinib** p**hase I/II (**13**)**	Treatment naïve ORR 71% (95% CI 48-89%), pretreated patients ORR 60% (95% CI 46-73%)	46%	4%

Abbreviations: FDA, Food and Drug Administration; MTC, medullary thyroid cancer; ORR, objective response rate; OS, overall survival; PFS, progression-free survival.

% patients requiring dose reduction or discontinuation due to adverse events or toxicity.

Vandetinib has one of the highest half maximal inhibitory concentrations against RET of the MKIs. Hence, it was evaluated in the ZETA study. Here, PFS of vandetinib compared with placebo was increased (HR 0.46; 95% CI 0.31-0.69) but, of note, there was no requirement to have progressive disease to participate (9). Follow-up studies with vandetinib have shown median PFS of 16.1 months (73). Treatment with cabozantinib in patients with progressive MTC was evaluated in the EXAM study with response rates of 28% observed. Of the patients on cabozantinib, 47.3% were alive at 12 months as opposed to 7.2% on placebo (10). Overall survival after long-term follow-up (minimum 42 months) in cabozantinib patients (harboring p.Met918Thr mutations) was 44.3 months vs 18.9 months for those on placebo (HR 0.60; 95% CI 0.38-0.94) (74). Treatment-related adverse events are frequent with this class agents, and include diarrhea, fatigue, hypertension, and other toxicities (75). Lenvatinib has had modest success in MTC with objective response rates (ORRs) of 22% to 36% and 1 study reported PFS of 9 months (95% CI 7 months, not evaluable) (11, 76). Since tolerated doses of these MKIs are usually well below that required for complete RET inhibition, their efficacy may depend on inhibiting VEGF receptors or other kinases (77). As such for those non-RET mutated advanced MTCs, the MKIs are currently the systemic treatment of choice. In contrast, for RET altered MTCs targeted specific RET kinase inhibitors have been successfully trialed in vitro (78, 79) and are finally in clinical trials and expanded access programs.

## Specific RET Inhibitors

RET-specific kinase inhibitors have the benefit of potent inhibition of the oncogenic driver with less off-target kinase inhibition and better patient tolerability. In the past few years, both selpercatinib and pralsetinib have been significant additions to the spectrum of treatment options for MTC (Table 2). In the clinical trials, both drugs were prescribed to patients who were naïve to MKIs as well as to those who had been pretreated. In patients with *RET* alterations, selpercatinib showed high ORR and rates of PFS at 1 year, irrespective of previous kinase inhibitor treatment (12). Those who had been pretreated showed an ORR of 69% (95% CI 55-81%) and rate PFS at 1 year of 82% (95% CI 69-90%). Treatment-naïve patients had slightly better responses with ORR 73% (95% CI 62-82%) and rate PFS at 1 year 64% (95% CI 37-92%). Of note, median PFS has yet to be reached. Dose reductions for toxicity were seen in 30% of patients with discontinuation rates of 2% (Table 2). While brain metastases in MTC are unusual, there appears to be a robust intracranial response with selpercatinib in non small cell lung cancer (NSCLC) and registered benefits in MTC case reports(80, 81). Pralsetinib in treatment-naïve patients showed an ORR 71% (95% CI 48-89%), and in pretreated patients an ORR 60% (95% CI 46-73%) (13). The effectiveness of these 2 targeted treatments after MKI therapy suggests maximal inhibition of the oncogenic kinase had not been reached with the more multitargeted therapies. Treatment-related adverse events were mild in the vast majority of patients on both drugs, with hypertension being the most common (17-21%). Dose reductions were seen in 46% of patients on pralsetinib mostly due to neutropenia and lymphopenia, and 4% of patients discontinued due to toxicity (Table 2). Ongoing surveillance has identified small bowel edema (82) and chylous effusions (83) as adverse effects of selpercatinib. Clinical trials exploring the role of these selective inhibitors in the neoadjuvant setting are ongoing and results may yield significant practice changes thereby providing more personalized options for patients (NCT04759911).

The patient experience of symptoms and toxicity has been notoriously absent from the literature. However, patient reported outcomes are now often integrated into clinical trials as their value and recognition is increasing. MTC patients on selpercatinib recorded clinically meaningful improvements in diarrhea and either maintained or improved health related quality of life scores (84). As such these selective inhibitors may be commenced predominantly for hormonal control in addition to structural disease.

## Systemic and Other Treatment Approaches

Exploiting the somatostatin receptors intrinsic to MTC has been trialed with somatostatin analogs in an attempt to modify disease progression. However there is little effect in disease control with no significant reduction of metastatic size or tumor markers (85). Adjuvant radiotherapy was thought to confer no significant benefit and is consistently not associated with an improved survival, but a recent systematic review suggested the risk of locoregional relapse can be minimized in high risk patients (86). Concern for tissue damage complicating future surgery is another consideration if pursing external beam radiotherapy. Traditional chemotherapy regimens (eg, dacarbazine and doxorubicin) are no longer recommended in MTC (5). Peptide receptor radionucleotide therapy (PRRT) has been explored in MTC due to their shared lineage with PRRT-responsive neuroendocrine tumors. Most are treated with somatostatin analogs radiolabeled with 90-yttrium and 177-lutetium (^177^Lu). ^177^Lu-DOTATATE has demonstrated some utility; 62% of metastatic MTC patients who were somatostatin receptor positive showed a radiological response, 51% had a symptomatic response and 51% a biochemical response (87). Overall, from this cohort, there were no complete responses but 10% had a partial response and 51% had stable disease. Among 220 patients, a systematic review described biochemical and objective responses of 37.2% and 10.6% in metastatic MTC patients on PRRT (88). In Hayes et al, 21 patients with metastatic MTC had PRRT with a median time to treatment failure of 14 months (95% CI 8-25 months) and a median overall survival of 63 months (95% CI 21, not reached) (54). They were unable to demonstrate a significant association between ^68^Ga-DOTATATE avidity and PRRT lengthening time to treatment failure but their numbers were small.

## Resistance to MKIs

**Figure 3.**
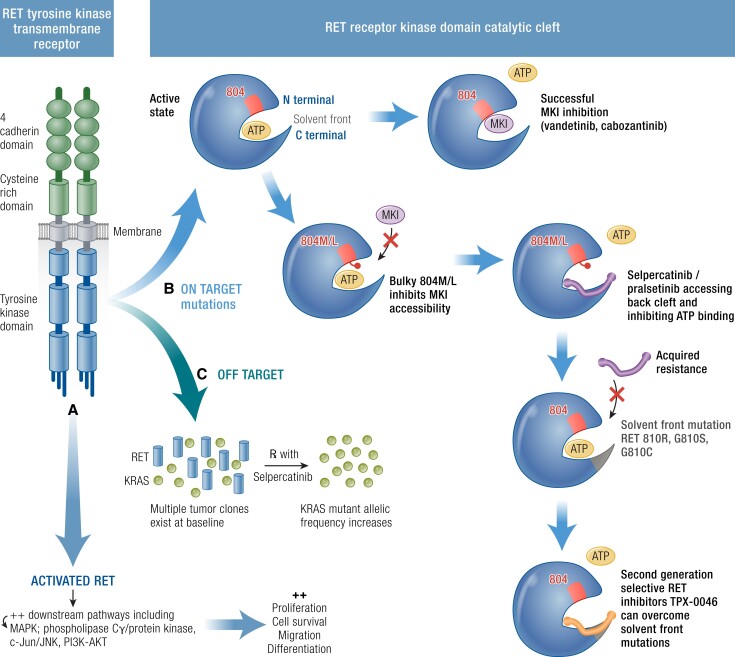
Mechanisms of MKI resistance. RET tyrosine kinase transmembrane receptor has constitutive activation due to RET point mutations leading to downstream pathway activation. (A) Resistance mechanisms are described. (B) On target: the RET receptor kinase domain catalytic cleft is activated when ATP causes phosphorylation. MKIs (vandetinib, cabozantinib) are able to hinder the ATP binding when there is no 804M/L mutation with bulky hydrophobic side chains. Selective RET inhibitors (pralsetinib and selpercatinib) avoid bulky inhibition by binding to the front and back clefts passing around the gate wall K578 residue to arrive at the back. Acquired solvent front mutations RET 810R, G810S, G810C hinder this binding rendering selpercatinib ineffective. Second-generation selective RET inhibitors TPX-0046 can overcome solvent front mutations. (C) Off Target mutations are shown with multiple tumor clones existing at baseline, reduction in RET, and subsequent increase in KRAS (or MET) allelic frequency.

Resistance to kinase inhibitors is the next challenge to overcome in the management of MTC. Acquired resistance occurs when patients have an initial clinical response to therapy and subsequently become resistant to therapy despite continuous treatment with the MKI. Clinical progression secondary to resistance can occur from selection pressure for clones with secondary somatic mutations. Resistance mechanisms are usually either on target; within the kinase domain itself, or off-target or bypass alterations, both of which are a concern for MKI use and selective RET kinase inhibitors utilized in MTC (Fig. 3).

## Mechanisms of Resistance

Within the RET kinase domain, steric inhibition of MKI binding is a form of “on-target” resistance. The kinase gatekeeper can limit access to the ATP binding pocket rendering it inaccessible. Vandetinib resistance is well known in MTCs harboring *RET* p.Val804Met. Here, replacement of valine 804 with longer hydrophobic side chain amino acids compromises inhibitor binding (Fig. 3) (89, 90). Selective RET inhibitors were developed to overcome gatekeeper resistance mutations and as such are effective for patients harboring *RET* p.Val804M/L alterations regardless of whether the mutation is germline, somatic or acquired. However, other on target mutations have been identified such as solvent front (p.Gly810) or hinge region (p.Tyr806) mutations after prolonged pralsetinib and selpercatinib use, limiting their continued efficacy in some patients (Fig. 3) (91, 92). Bypass pathway activation is another resistance mechanism following continuous MKI use. Despite inhibition of the primary oncogenic kinase driver subclones within the tumor driven by other oncogenic alterations can emerge. Following selpercatinib and pralsetinib use, *MET* and *KRAS* amplification have been identified in *RET* driven NSCLC (93).

## Identification of Resistance

Liquid biopsies of ctDNA (circulating tumor DNA) are a potential tool to identify resistant clones early to enable recognition of resistance prior to radiological or clinical progression. From a simple blood sample, circulating cell free DNA can be assessed and is utilized in oncology predicting disease progression in breast and colorectal cancers, and its utility in thyroid cancer is growing—reviewed in (94). Early identification of resistance mutations through ctDNA techniques may allow clinicians to prepare for further line therapies. However, the yield in MTC can be challenging with 1 cohort of 50 MTC patients harboring *RET* p.Met918Thr alterations only yielded a positive ctDNA result via digital droplet polymerase chain reaction in 32% (95). An association with higher levels of ctDNA and poor outcomes has been identified showing its utility in prognostication as well as potentially treatment response and diagnostics (94).

## Paraneoplastic Syndromes

Excess calcitonin secretion from metastatic tumors can be challenging to manage. Fortunately, kinase inhibitors usually elicit prompt biochemical responses (32), and debulking metastatic disease where possible may also help symptom palliation. Medically managing refractory diarrhea with first-line therapy loperamide is often unsuccessful, and there are mixed results reported with somatostatin analogs (96). Glycopyrrolate in octreotide resistant diarrhea has had success in 1 case report (97), but the authors find ondansetron to be most effective and utilize it as first line (98).

Cushing syndrome due to ectopic adrenocorticotropin is a rare paraneoplastic complication occurring in 0.7% of MTC cases. It is associated with increased mortality together with significant morbidity from weight gain, abdominal striae, myopathy, and lethargy (2). Antiadrenal therapies (medical or bilateral adrenalectomy) may be warranted if surgical tumor debulking is not feasible or incomplete. Kinase inhibitors including selpercatinib (99) and vandetinib (100) can result in dramatic normalization of cortisol and adrenocorticotropin in addition to improving disease control. If utilizing multiple pharmacological agents, cautious ECG monitoring for QT prolongation is warranted due to similar side effects of ketoconazole, vandetinib, and mitotane.

## Future Directions

There are promising new therapies for MTC already in development. Importantly, the next generation of RET-specific inhibitors designed to overcome on-target acquired resistance are already in first in human phase I study (NCT04683250; NCT05241834). Other new directions include study of microRNA, cyclin-dependent kinases, and genetically engineered T cells. MicroRNAs (small noncoding RNAs) are a burgeoning area in cancer research in their role of RNA silencing and post-transcriptional regulation of gene expression. Future studies may be able to show their role in staging as there are data showing how they promote chemoresistance and may predict prognosis. miR-375 expression is a negative prognostic marker for MTC (101) and miR-153-3p is a RET-regulated tumor suppressor in MTC (102). Preclinical studies have shown potential in genetically engineered T cells targeted to CEA, calcitonin and *RET* p.Met918Thr to treat metastatic MTC (103). Cyclin-dependent kinase 5 has been associated with cancer development and cyclin-dependent kinase 5 inhibitors block the growth of tumors in distinct models of MTC but has yet to be explored in clinical trials (104). Chimeric antigen receptor T cells are also under investigation in a Phase I study for MTC (NCT04877613).

Prostate-specific membrane antigen (PSMA) is used as an imaging target for prostate cancer (^68^Ga-PSMA-PET CT), but PSMA is now known to be expressed in other cancer types including thyroid. Case reports have shown PSMA-PET scans identifying metastatic MTC, and PSMA-targeted radioligand therapy may be a future therapeutic target (105, 106). Other PRRT options include fibroblast activation protein inhibitor, a ubiquitous oncological target, which in 1 case report had a mixed response when ^177^Lu-DOTAGA (SA.FAPi)_2_ was administered as a last line of therapy to a patient with MTC (107).

Immunotherapy has heralded success in many cancers, particularly those with high tumor mutational burden. The immune landscape of MTC was characterized by Pozdeyev et al and showed the presence of an immune infiltrate was much more prevalent than had been previously described (108). MTCs may have considerable PD-L1 expression suggesting pembrolizumab may be a further avenue for treatment options (109). Several clinical trials are ongoing involving immunotherapy alone (NCT03072160, NCT03246958) or in combination for therapeutic strategies for MTC.

## Conclusions

Novel diagnostic and therapeutic modalities have changed the way clinicians approach advanced MTC over the past decade. Nonetheless, cure remains possible only with early diagnosis followed by surgery. Germline RET testing in MEN 2 and 3, and somatic RET testing in sporadic MTC have revolutionized the treatment options available to patients. A new histological grading system has been introduced which better predicts prognosis at the time of diagnosis. Functional imaging can improve detection and characterization of metastatic disease. Phase I/II clinical trials of selective RET inhibitors have made highly effective and tolerable therapy available to patients with advanced MTC. Acquired resistance to kinase inhibitors is emerging as a new challenge for some patients, and novel strategies, including second-generation RET inhibitors and other treatment approaches, are already in development.

## Disclosures

M.G.: Nil. R.C.B.: Advisory Board Amgen, Eisai Inc, Kyowa Kirin, Ipsen, Speaking honoraria Kyowa Kirin, Amgen, Eisei Inc. B.R.: Advisor to Eisai Inc, Exilixis and Lilly. Investigator for Lilly and Exilixis. M.G. has nothing to declare. R.C.B. has been on the advisory board and had honoraria received from Eisai Inc Amgen, Kyowa Kirin, Ipsen. B.R. has been an advisor to Eisai Inc, Exilixis and Eli Lilly and Co. L.W. has received compensation for Bayer HealthCare Pharmaceuticals, Coherus BioSciences, Curie Therapeutics, Eli Lilly and Co, Eisai Inc, Exelixis, Genentech USA, Morphic Therapeutics, Honoraria received for data safety monitoring board: PDS Biotechnology, Research support from: Eisai Inc. Eli Lilly and Co. Molecular Templates, Inc. Regeneron.

## Abbreviations

CCK: cholecystokinin
CEA: carcinoembryonic antigen
CT: computed tomography
DT: doubling time
MEN: multiple endocrine neoplasia
MKI: multikinase inhibitor
MRI: magnetic resonance imaging
MTC: medullary thyroid cancer
ORR: objective response rate
PET: positron emission tomography
PFS: progression-free survival
PRRT: peptide receptor radionucleotide therapy
PSMA: prostate-specific membrane antigen
RET: rearranged in transfection
